# Treatment of Emergencies

**Published:** 1887-07-02

**Authors:** 


### Till the Doctor Comes.

[Inquiries and Communications for this column should be addressed "Physician," care of the Editor of The Hospital.]

### XXI.—TREATMENT OF EMERGENCIES.

(*Continued.*)

*Bleeding.*—When an artery of any size is wounded, the blood comes out in jets, and is bright red: bleeding from a vein, on the contrary, oozes out, and is much darker in colour. The following are the usual means adopted to stop bleeding:—

1. *Direct pressure.*—By means of the finger pressed firmly on the wounded spot, serious loss of blood need in no case occur, if the bleeding spot can be seen: it may always be thus controlled. Persons must remember this, and never lose their presence of mind. By strips of lint folded into a thick pad, or a towel folded and firmly pressed on the place, either by the hand or by a firm bandage, bleeding from the head can always be controlled, for the pad presses the vessel firmly against the bone and closes it. In any position where the vessel can be thus compressed against a firm substance this will succeed, but the pad must not be removed for forty-eight hours, unless absolutely necessary for other reasons, or the bleeding will recommence. In some cases, where blood comes from the bottom of a large deep wound, a sponge or some strips of lint may be firmly packed into the wound, and kept there; but this will require some courage to do effectually, as it causes much pain, and other means should be first tried.

2. *By what is known as a tourniquet.* A temporary one may be thus applied. Take a handkerchief and tie it tightly round the limb above the bleeding spot; introduce under this a firm piece of wood, and twist it round and round, so as to tighten the handkerchief till all bleeding stops. This plan is very useful when the bleeding is from some part of one of the limbs, but is now usually superseded by the following:—

3. *By tying an elastic band firmly round the limb.*—This is much more easily applied. At all railway stations these bands are now kept, and the officials of the railway company instructed as to their use If one is not kept in every house, there ought to be one in every village, as it is invaluable when a large vessel of the limb is wounded. In applying it, it is simply wound two or three times round the limbs above the bleeding point, being drawn as firmly as possible each time, and it is then tied securely, or is made to fasten with two hooks. This proceeding renders unnecessary detailed directions as to the course of the different arteries, and will effectually control any bleeding from a wounded artery in the limbs. Care must be taken to draw the band sufficiently tight, and it must be looked on as only a temporary means of stopping severe hæmorrhage from an artery till surgical assistance can arrive. It remains to notice a few special forms of bleeding.

*Bleeding from the Nose.*—Do not let the patient hang his head over a basin; let him sit upright and apply cold to the bridge of the nose and nape of the neck, or syringe ice-cold water up the nose. Hold the hands above the head. Do not blow the nose. If these means fail the nose must be plugged by a surgeon.

*Bleeding from Varicose Veins of the Leg.*—Raise the leg considerably above the level of the body, keep the patient at full length on a sofa, put a pad and bandage over the wounded part, and apply firm pressure in this case *below* the wound, because the course of blood in the veins is from below upwards towards the heart.

(*To be continued.*)

